# New and Cheap Method of Ventilation, Applicable to Any Apartment

**Published:** 1862-10

**Authors:** T. S. Lambert

**Affiliations:** Peekskill, N. Y.


NEW AND CHEAP METHOD OF VENTILATION, APPLICABLE TO ANY APARTMENT.—*Messrs. Editors*:—It was not my intention to write the following paper until the results of experiments now progressing should enable me to make it more definite and conclusive. Your remark that the summer is upon us, and that suggestions might induce experiments and applications by others, has made me reconsider my intention, and accede to your request. All immaturity of ideas, or expression of them, will therefore be excused, and attributed to a desire to benefit, if possible, the poor fellows who are sacrificing their lives for their country's good.

Ventilation has hitherto been chiefly considered with reference to properly supplying the requisite amount of oxygen to the air of rooms. Attention has also been drawn to the importance of moistening it, and removing the exhalations from the skin. Some writers have properly allowed an extra movement of air through a room in order to carry off these exhalations.

In all cases, some points of essential importance seem to have been overlooked, partial views only having been taken of the conditions of the air essential to its best use, either by the well or sick, under certain circumstances. While it has been thought worth while to increase the moisture of too dry air, no means seem to have been sought for drying the air when too moist, without at the same time elevating the temperature, already too high; nor have any means been sought for changing the air about us from a negative to a positive electric condition; and while the electric machine has been called in to charge a person with the electric fluid, the great electrical changes in the atmosphere, wrought by the Creator on a grand scale by the summer shower, and either the cause of, or attendant upon, the invigorated condition of all things that live, has not been attempted to be imitated on a small scale by man.

Partial views only having been taken of the necessary conditions to be obtained, only partial means have been employed for gaining what has been attempted.

Whether the plenum or the vacuum method of ventilation is the better, has been much discussed. Machines for both kinds of work, and fires adapted to do both, have been invented, and work with great success, except in certain kinds of weather: to-wit, when the temperature of the atmosphere is elevated, and it is also saturated with moisture, "hot, muggy, dog-day weather;" when the dew-point is high, and the air, of course, negatively electric. In such cases all means used by man have hitherto failed to produce the conditions of the air desirable for sick or well, and which have alone heretofore been wrought on a grand scale by natural ventilation. To imitate this should be the effort of man. This failure in producing the conditions most favorable to health and comfort, has doubtless been caused by failing carefully to observe what these conditions are.

First, oxygen in proper proportion to the air must be maintained. For this purpose a certain quantity of air must be passed through any occupied room.

Secondly, the exhalations from the skin and lungs must not be removed too rapidly, nor, on the other hand, be allowed to accumulate by being removed too slowly. Hence, if the air is too dry, it must be moistened, and *if it is too moist, it must be dried*, either with or without raising its temperature, as the case may be.

Thirdly, the body and mind being always most active when the atmosphere is positively electric, this condition must, if possible, be produced.

Now, there is a unity in this trinitarian ventilation, for in cases heretofore difficult, viz., in hot, damp weather, the means adapted to gain one end will gain all three, which triple unity is one of the strongest theoretical proofs of the correctness of the ground taken. The means consist simply in *icing the air that flows into the room*, or is in it.

When the temperature of the atmosphere is nearly the same as that of the body, the vacuum effect of its heat is very little, and the vacuum effect of a fire in a vacuum chimney is lessened, and of course has no effect on the dew-point of a nearly saturated atmosphere; hence the viscous perspiration remains on the skin, the blood is unrelieved, the brain is oppressed, and the electric condition of the air and the body are unimproved, however great may be the quantity of such air poured through the room by any plenum machine.

Let, now, the air at the plenum openings properly located, be iced, the pressure of the cold or iced air will send the heated air up; the air flowing in over the ice will of course deposit a part of its moisture, and when again warmed and expanded by contact with persons and things in the room, will absorb the exhalations from the skin and lungs, and becoming also positively electric, will feel like the delightful atmosphere of summer after a shower.

Are these suggestions practical? Most certainly they are, for it was the practical that suggested the experiments that led to these theoretical views, accident having induced a series of experiments and thoughts that were the basis of what has been said. In fact, the chief cause of the delight these thoughts awaken is the unity, simplicity and practicability of the ideas suggested.

I can not, from the number of experiments yet tried, say what detailed method will be the best for gaining the desired object, and I doubt not that the ingenuity of others will excel any method I can now or shall hereafter suggest.

For a simple, convincing experiment, merely a box, large enough to hold a dozen or twenty pounds of ice, is all that is required. Let some holes be cut in one side near the bottom. It needs no cover. Let it be placed under the sash of a window, so that two or three inches of it shall be outside the window; and, if the box is not as long as the window is wide, the opening under the sash at the ends of the box should be closed. Thus the air can pour through the box and ice into the room. A vessel of sufficient size should be placed beneath the box to catch the drip. The higher up in the room the box is placed, the better. So, if the upper sash drops, the box can be placed on it. It would, of course, be still better to have the ice above the room, and the air flow over the ice through several openings into the room.

Such a box was placed under the lower sash of a window in a room containing about 600 cubic feet of air. In the box I placed eleven pounds of ice, in three lumps, at half-past one, P. M. Mercury 92, north side of house; inside 90. In one hour, it had fallen to 84; in two hours, to 76. Outside it stood at 90. At ten, it was 78 inside, ice not quite gone. Thermometer, being in the middle of the room, placed near the floor it would fall, raised to ceiling it would rise. If I remained in the room half an hour, it would rise about three degrees.

On another day, twenty pounds of ice, in eight lumps, were placed in the box at 9, A. M. At 10, the mercury stood at 78 in the room, 86 outside. It did not go above 78, whether I was in the room or not; and next morning, at 9, twenty-four hours after the ice was placed in the box, it having not yet melted, the mercury stood at 69 in the room; at 80 in an adjoining room, and had been up to 90 there in the time of the experiment.

The door of this room, opposite the window, was so far from tight that no special vacuum opening was thought necessary; there was a direct opening over it. That it and the washboard would let so much cold air flow out as they did, was objectionable. A smoke being made by burning paper in the room, none of it was observed to pass through the box out of the window, but soon the circulation of air in the room and through the box inside the window was very beautifully exhibited, curling over the ice, then tumbling down in miniature cascades, sweeping along the floor, then eddying up to be again warmed and again pass down, drawn through the box by the falling iced air.

The feeling of the air thus iced had none of the dankness of a cellar, but was invigorating to the lungs and delicious to the skin, suggesting every moment that iced air is one of the materia medica, as well as one of the mechanical forces that shall prove a balm to many a fevered body. It was entirely suggestive of the after summer-shower atmosphere, that is so delightful to the well and so healing to the sick. What are the polar regions but one vast ice box, with bergs for lumps, through which the air circulating is cooled, depositing its moisture and thence pouring down upon us from the northwest with healing in its wings—more invigorating than any cordial, more healthfully stimulating than any dram, more cleansing than any bath, and more soothing and soporific than any opiate. With the air properly iced, all "nights will be found good for sleeping."

With proper arrangements, the rooms of sick or well persons can be ventilated with air of any desirable temperature, and I feel certain that hospitals, either temporary or permanent, can be constructed for such purposes without any cost additional to that now made, and that the same may be true of the dwellings of the rich or poor, while the ice required for such ventilation would cost but a trifle. An ordinary house, properly arranged, could be thus ventilated with the expenditure of from fifty to one hundred pounds of ice, costing from ten to twenty-five cents.

To have the experiments most successful, the doors should be made tight with rubber, list or the like, and a proper opening in the upper part of the room should be made to let off the air. The windows should be in some way doubled, as, in this case, they let in heat, as in winter they let it out. A curtain, sheet or blanket can be fastened to the frame of the window, or "double windows" of glass or paper can be added —Hunt's patent double windows being the best for this purpose of any which I have seen, since they go on inside, can be partly or wholly glazed with paper, can be easily taken off and put on, are neat as well as convenient, and made at half or two-thirds the usual expense. The walls also should, as far as possible, be non-conducting; hence papering them will make the experiment more successful.

When the experiments now progressing shall be completed, I will inform you of the results; till when, hoping that these remarks will suggest something practical to the ingenious minds of your many readers,

I am respectfully yours, T. S. Lambert.

*Peekskill, N. Y., July*, 1862. [*Med. & Surg. Jour.*

